# Commentary: Effect of cochlear implantation on vestibular function in children: A scoping review

**DOI:** 10.3389/fped.2022.1101540

**Published:** 2022-12-21

**Authors:** M. Yong, E. Young, J. Lea, H. Foggin, E. Zaia, F. K. Kozak, B. D. Westerberg

**Affiliations:** ^1^ Division of Otolaryngology - Head and Neck Surgery, University of British Columbia, Vancouver, BC, Canada; ^2^ Southwest Health, Warrnambool, VIC, Australia; ^3^ Division of Otolaryngology - Head and Neck Surgery, St. Paul's Hospital, Vancouver, BC, Canada; ^4^ Audio-Vestibular Clinic, Vancouver, BC, Canada; ^5^ Division of Otolaryngology - Head and Neck Surgery, British Columbia Children's Hospital, Vancouver, BC, Canada

**Keywords:** cochlear implant, vestibular function, scoping review, pediatric otolaryngology, otolaryngology

**A Commentary on:**
Effect of cochlear implantation on vestibular function in children: A scoping review By Yong M, Young E, Lea J, Foggin H, Zaia E, Kozak FK, Westerberg BD. (2022). Front. Pediatr. 10:1101540. doi: 10.3389/fped.2022.1067453

## Commentary

This recent scoping review on the effects of cochlear implantation on vestibular function in children, published in September 2022 by Gerdsen et al. (1), follows our group's own previous systematic review on the same topic, published in 2019 in the *Journal of Otolaryngology – Head and Neck Surgery* (2). Despite their comprehensive literature review, the authors did not cite our similar review which included many of the same references; a discussion on the differences in the evaluation, results, and conclusions between the two papers would have made an interesting addition, and we feel that further discussion of the analyses is a necessary addition to this area of research. Overall, we agree that the effect of cochlear implantation on objective and subjective vestibular findings in children is largely understudied and poorly understood.

Accordingly, we wish to compare and contrast the findings of this updated review to the findings of our 2019 systematic review. Firstly, the inclusion criteria for both studies were similar, examining children under the age of 18 who received cochlear implantation and had pre- and post-operative vestibular testing performed. Gerdsen et al. (2022) included a total of 14 relevant studies compared to the 11 studies included in our previous analysis. The inclusion of four new studies, one case series and three cohort studies, represents an updated review of the literature during the intervening three years (3–6). However, two of the studies included in our 2019 analysis were excluded in the recent review, one of which was likely excluded due to the publication being a dissertation and the other excluded for unknown reasons (7, 8). Lastly, one study which was excluded in the 2019 analysis due to difficulties with incorporating the data in the meta-analysis was included in the 2022 analysis (9).

With regards to the study outcomes, there was no updated meta-analysis performed by Gerdsen et al. (2022) to compare to our previous study. We recognize the authors’ concerns regarding the heterogeneity of studies to date and that a meta-analysis may include an unknown degree of bias. Our meta-analysis findings in 2019 did support that cervical vestibular-evoked myogenic potentials (cVEMPs) undergo a statistically significant change after cochlear implantation. This agrees with Gerdsen et al. (2022) with regards to their qualitative cVEMP findings. Concerning caloric testing, our review showed that while one individual study by Gupta et al. (2017) showed a decrease in caloric responses after implantation, the pooled analysis did not show any statistically significant difference (10). The qualitative analysis of caloric testing by Gerdsen et al. (2022) generally reflects our own findings that the majority of studies did not identify a significant decrease in caloric responses. However, their review adds an additional paper by Guan et al. (2021), which also notes a statistically significant decrease in caloric responses (3). The impact of this additional study on pooled risk for decreased caloric responses is unknown, as an updated meta-analysis was not repeated with this new data.

Gerdsen et al. (2022) provided a further exploration of other vestibular testing, including rotary chair, ocular-evoked myogenic potentials (oVEMPs), and video head impulse testing (vHIT). Their review demonstrates that, based on available data, rotary chair and vHIT do not show any significant alterations after implantation. However, they found that the literature supported an impairment of oVEMPs, highlighting that utricular dysfunction may also accompany saccular dysfunction after implantation. While this may be the case in some individual studies, a study by Li et al. (2020) indicated that oVEMPs recover after an initial deterioration in function, making it difficult to conclude whether oVEMPs are significantly affected by implantation (6). Our review found a general lack of data on rotary chair, vHIT, and oVEMPs, which precluded a quantitative analysis.

Importantly, Gerdsen et al. (2022) also found that significant heterogeneity exists in the literature, rating all studies as moderate or high risk of bias. These findings were based on differences in outcome measurements which made comparison across studies difficult to interpret. This mirrors our own review findings, where we also highlighted the need for standardization of objective and subjective perioperative vestibular testing in children.

Overall, Gerdsen et al. (2022) offer an updated qualitative review on the topic of vestibular outcomes following pediatric cochlear implantation. Given the significant heterogeneity still found in the literature since 2019, standardization of outcome measurement in the perioperative pediatric cochlear implantation period is needed in order to conduct a more clinically meaningful meta-analysis. At present, no conclusion on vestibular impairment from cochlear implantation in children can be drawn.
